# Association Between the Dose of Tofacitinib and Risk of Herpes Zoster in Patients With Rheumatoid Arthritis: Analysis of Japanese Adverse Drug Event Report Data

**DOI:** 10.7759/cureus.62372

**Published:** 2024-06-14

**Authors:** Kazumasa Kotake, Satoru Mitsuboshi

**Affiliations:** 1 Pharmacy Department, Zikei Hospital/Zikei Institute of Psychiatry, Okayama, JPN; 2 Pharmacy Department, Kaetsu Hospital, Niigata, JPN

**Keywords:** japanese adverse drug event report database, janus kinase inhibitor, herpes zoster, rheumatoid arthritis, dose of tofacitinib

## Abstract

Background: Tofacitinib is one of the Janus kinase inhibitors approved for the treatment of rheumatoid arthritis. The major adverse event of this drug is herpes zoster, which can lead to death in severe cases. The risk of herpes zoster has been studied at 10 mg/day of tofacitinib; however, 5 mg/day, which is recommended in patients with chronic kidney disease, is unclear.

Objective: To investigate whether 5 mg/day of tofacitinib reduced the risk of herpes zoster compared with 10 mg/day in rheumatoid arthritis patients.

Methods: We analyzed the Japanese Adverse Drug Event Report Data (JADER) database and compared the frequency of herpes zoster in rheumatoid arthritis patients treated with tofacitinib 5 mg/day and 10 mg/day. Multivariable logistic regression analysis was performed to identify the risk factors for herpes zoster in tofacitinib users.

Results: A total of 812 tofacitinib users with rheumatoid arthritis were identified, including 131 with herpes zoster. Disproportionality for herpes zoster was observed between 5 mg/day and 10 mg/day (reporting odds ratio (OR): 0.68, 95% confidence interval (CI): 0.47-0.98, P = 0.045). Multivariable logistic regression analysis showed that the risk of herpes zoster was significantly increased in female patients (OR: 1.87, 95% CI: 1.12-3.12, P = 0.016) and methotrexate users (OR: 1.69, 95% CI: 1.12-2.54, P = 0.013) and significantly decreased with tofacitinib 5 mg/day compared with 10 mg/day (OR: 0.62, 95% CI: 0.40-0.96, P = 0.032).

Conclusion: We suggest that tofacitinib 5 mg/day may decrease the risk of herpes zoster compared with 10 mg/day in rheumatoid arthritis patients.

## Introduction

Tofacitinib is a Janus kinase inhibitor approved for the treatment of rheumatoid arthritis [1]. It is used in patients who are unable to achieve remission or low disease activity with prednisolone or methotrexate [2]. However, because tofacitinib blocks cytokine signaling and modulates the immune response, it increases the risk of various infections [3].

Herpes zoster is an acute viral infection caused by the reactivation of the varicella-zoster virus. It is a major adverse event associated with tofacitinib, with a reported incidence rate of 2.5 per 100 patient-years [4]. The onset of herpes zoster can lead not only to interruption of therapy for rheumatoid arthritis and increased risk of hospitalization but also to death in severe cases [5,6]. Thus, reducing the onset of herpes zoster in patients using tofacitinib may improve patient outcomes. Reported risk factors for herpes zoster associated with tofacitinib are sex, age, concomitant use of prednisolone or methotrexate, and tofacitinib dose [5,7,8]. In particular, a 2.5-fold lower incidence of herpes zoster was reported in patients treated with 10 mg/day compared with 20 mg/day [9], suggesting that a further dose reduction of tofacitinib may further reduce the risk of herpes zoster.

A reduction of tofacitinib from 10 mg/day to 5 mg/day has been recommended for patients with chronic kidney disease (CKD), hepatic impairment, or those using drugs that inhibit cytochrome P450 3A4 [10]. Notably, these patients were able to maintain remission and low disease activity even after this dose reduction in the clinical setting [11]. Furthermore, the number of patients with rheumatoid arthritis has been increasing as the population ages [12], and about 20% of these patients have CKD [13]. Although there are many reasons to reduce the dose of tofacitinib, it remains unclear whether the risk of herpes zoster is reduced when the dose is adjusted from 10 mg/day to 5 mg/day. We investigated whether a dose of tofacitinib at 5 mg/day reduced the risk of herpes zoster compared with 10 mg/day in patients with rheumatoid arthritis using the Japanese Adverse Drug Event Report (JADER) database.

## Materials and methods

Data source

The JADER database is a spontaneous reporting system for drug-related adverse events in Japan, provided by the Pharmaceuticals and Medical Devices Agency (PMDA), the Japanese regulatory authority. The database comprises four tables:

DEMO: Contains patient demographic information

DRUG: Contains drug information and categorizes drugs into three types: "suspected drug," "concomitant drug," and "interacting drug"

REAC: Contains information on adverse events

HIST: Contains information on primary diseases

Data collection

Data recorded from April 2004 to February 2021 were downloaded from the PMDA website (http://www.pmda.go.jp/) on June 26, 2021.

Study design

This retrospective, observational cohort study analyzed patients enrolled in the JADER database. Publicly accessible at http://www.pmda.go.jp/, the JADER database contains over 500,000 case reports of potential drug adverse events reported in Japan since 2004. Studies utilizing the JADER database have been proposed to investigate potential associations between drugs and adverse drug reactions, focusing on signal detection [14-16].

Statement of ethics

The JADER database is a publicly available database, and since it uses anonymized patient data, informed consent was not required. The study was conducted in accordance with the ethical standards laid out in the 1964 Declaration of Helsinki.

Inclusion criteria

All tofacitinib users with rheumatoid arthritis treated with 5 mg/day or 10 mg/day were included, regardless of whether the data reporter suspected a possible adverse event.

Collected variables

We downloaded data with permission from the PMDA website (https://www.pmda.go.jp) on June 26, 2021, which included 693,301 patient records reported between April 2004 to February 2021. Data on sex, age, body weight, use of prednisolone or methotrexate, and presence of CKD were also collected. Only oral prednisolone use was included because intravenous administration is generally used for a limited duration. The use of methotrexate was defined as oral administration for the treatment of rheumatoid arthritis. Even if data on the route of administration or purpose were missing, 16 mg/day or less of methotrexate (this is the maximum approved dose for rheumatoid arthritis in Japan) was also defined as the use of methotrexate. CKD was defined as reporting the Preferred Terms “chronic kidney disease”, “kidney failure”, and “dialysis” as a comorbidity in the JADER database based on a previous study [17].

Outcome definition

We defined outcome as reporting the preferred term “herpes zoster” based on a previous study [18].

Statistical analysis

First, the chi-square test was used to compare the frequency of herpes zoster between tofacitinib doses of 5 mg/day and 10 mg/day. The reporting odds ratio (ROR), which can be generally used to identify drug-associated adverse events [19,20], 95% confidence interval (CI), and P values were calculated. Second, multivariable logistic regression analysis was performed to identify the risk factors for herpes zoster in tofacitinib users, and the adjusted OR, 95% CI, and P values were calculated. Age was stratified into ≤ 60 years old and > 60 years old, and body weight into ≤ 40 kg and > 40 kg. Sex, stratified age groups, stratified body weight, concomitant use of prednisolone or methotrexate tofacitinib dose, and presence of CKD were included in the analysis. All analyses were performed using R version 4.1.0 (R Foundation for Statistical Computing, Vienna, Austria). A P value < 0.05 was considered statistically significant.

## Results

**Figure 1:**
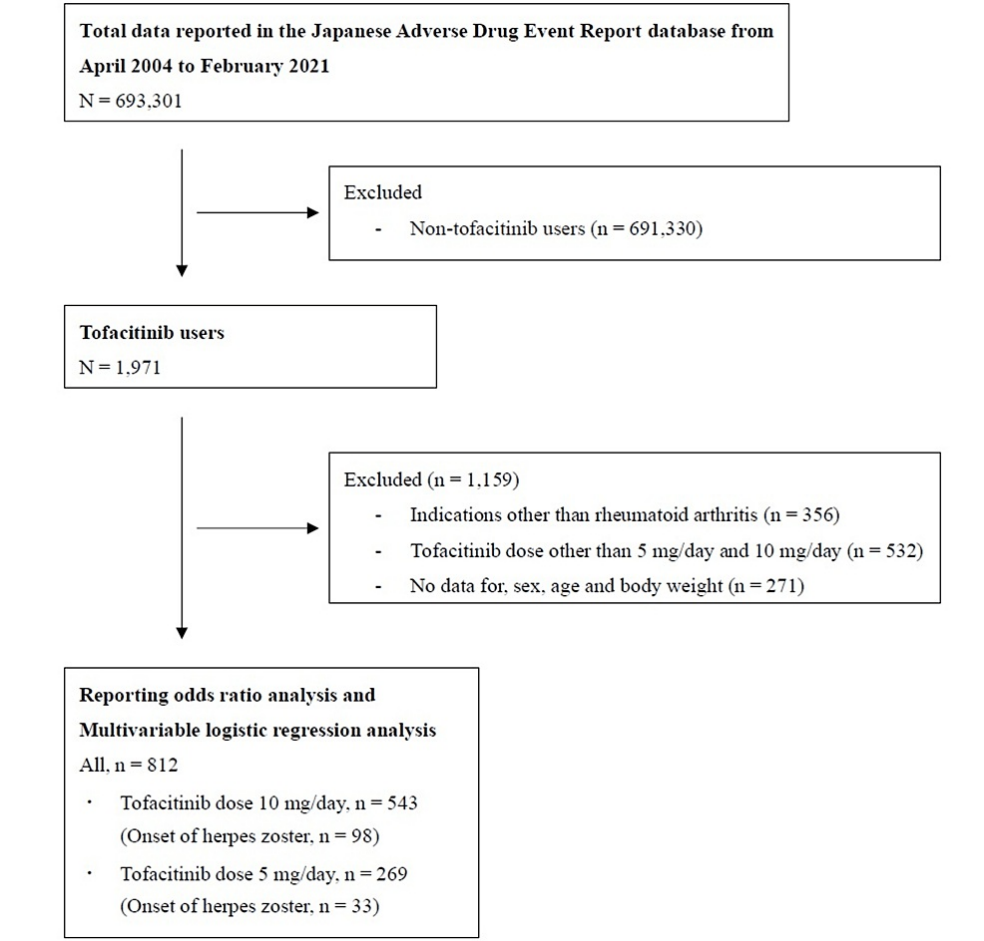
Flow chart of the patient selection process

We collected data on 693,301 patients from the JADER database. Among them, there were 1,971 tofacitinib users. Out of these, 812 were included in the study after excluding 1,159 who met the exclusion criteria. The flow chart of the patient selection process is shown in Figure 1.

Of 812 tofacitinib users identified in the database, herpes zoster was reported in 131. As shown in Table 1, disproportionality for herpes zoster was observed between doses of 5 mg/day and 10 mg/day (ROR: 0.68, 95% CI: 0.47-0.98, P = 0.045).

**Table 1:** Proportions of herpes zoster in patients with rheumatoid arthritis according to the tofacitinib dose *Chi-square test. A P value < 0.05 was considered statistically significant.

	Onset of herpes zoster/total, n (%)	Reporting odds ratio (95% confidence interval)	P value*
Tofacitinib dose 10 mg/day	98/543 (18)	1 [Reference]	
Tofacitinib dose 5 mg/day	33/269 (12)	0.68 (0.47–0.98)	0.045

**Figure 2:**
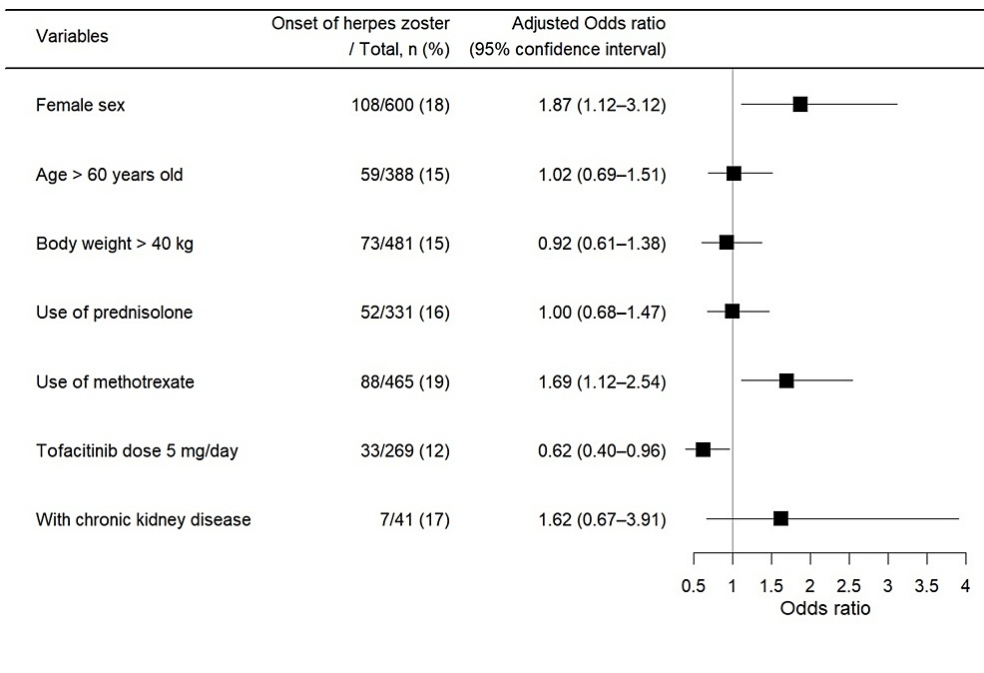
Forest plots for multivariable logistic regression analysis

As shown in Table 2 and Figure 2, the multivariable logistic regression analysis showed a significantly increased risk of herpes zoster in female patients (OR: 1.87, 95% CI: 1.12-3.12, P = 0.016) and methotrexate users (OR: 1.69, 95% CI: 1.12-2.54, P = 0.013), and significantly reduced risk with a tofacitinib dose of 5 mg/day compared with 10 mg/day (OR: 0.62, 95% CI: 0.40-0.96, P = 0.032).

**Table 2:** Multivariable logistic regression analysis of potential risk factors for herpes zoster in tofacitinib users *Multivariate logistic regression analysis. A P value < 0.05 was considered statistically significant. Modeling was based on a complete case analysis.

	Onset of herpes zoster/total, n (%)	Odds ratio (95% confidence interval)		P value*
		Crude	Adjusted	
Male sex	23/212 (11)	1 [Reference]	1 [Reference]	
Female sex	108/600 (18)	1.80 (1.12–2.92)	1.87 (1.12–3.12)	0.016
Age ≤ 60 years old	72/424 (17)	1 [Reference]	1 [Reference]	
Age > 60 years old	59/388 (15)	0.88 (0.60–1.28)	1.02 (0.69–1.51)	0.935
Body weight ≤ 40 kg	58/331 (18)	1 [Reference]	1 [Reference]	
Body weight > 40 kg	73/481 (15)	0.84 (0.58–1.23)	0.92 (0.61–1.38)	0.677
No use of *prednisolone*	79/481 (16)	1 [Reference]	1 [Reference]	
Use of *prednisolone*	52/331 (16)	0.95 (0.65–1.39)	1.00 (0.68–1.47)	0.987
No use of methotrexate	43/347 (12)	1 [Reference]	1 [Reference]	
Use of methotrexate	88/465 (19)	1.65 (1.11–2.45)	1.69 (1.12–2.54)	0.013
Tofacitinib dose 10 mg/day	98/543 (18)	1 [Reference]	1 [Reference]	
Tofacitinib dose 5 mg/day	33/269 (12)	0.64 (0.42–0.97)	0.62 (0.40–0.96)	0.032
Without chronic kidney disease	124/771 (16)	1 [Reference]	1 [Reference]	
With chronic kidney disease	7/41 (17)	1.07 (0.47–2.48)	1.62 (0.67–3.91)	0.281

## Discussion

To our knowledge, this is the first report to suggest that a dose reduction of tofacitinib from 10 mg/day to 5 mg/day may further reduce the risk of herpes zoster. The number of patients with rheumatoid arthritis complicated by CKD is increasing [13], and a tofacitinib dose of 5 mg/day may be indicated for many of them. The clinical benefit of tofacitinib 5 mg/day for rheumatoid arthritis has been reported previously [11]; therefore, an appropriate dose reduction of tofacitinib in patients at high risk of herpes zoster may mitigate the risk of reactivation. A network meta-analysis examining the risk of herpes zoster with Janus kinase inhibitors in patients with immune-mediated inflammatory diseases, including rheumatoid arthritis, also showed a lower risk of herpes zoster with lower doses of tofacitinib, which supports our results [21]. In this way, interruptions in the treatment of rheumatoid arthritis could be avoided, which could lead to improved patient outcomes.

Female sex and concomitant use of methotrexate were also suggested as risk factors for herpes zoster in tofacitinib users, aligning with previous studies. It has been reported that the female sex might be more likely to have a higher prevalence of risk factors for herpes zoster, but the reasons are unclear [22]. Methotrexate has been reported to increase the incidence of herpes zoster in rheumatoid arthritis patients when used in combination with tofacitinib [7]. Greater inhibition of cytokine signaling with concomitant use of these drugs may explain this increased risk [23].

Limitation

Analyses using spontaneous reporting systems, such as the JADER database, have some notable limitations [24]. First, entries with missing data regarding tofacitinib dose were excluded because we were comparing doses of 5 mg/day and 10 mg/day. Given that the usual dose of tofacitinib is 10 mg/day, it is possible that the excluded data included more data for 10 mg/day [25]. Thus, the actual ratio of herpes zoster incidence between the tofacitinib dose of 5 mg/day and 10 mg/day may have been distorted. A similar distortion could also occur for missing data on sex, age, and body weight. Second, although the JADER database contains data that allow for adjustment of covariates (sex, age, body weight, and presence of CKD), other comorbidities, such as the presence of human immunodeficiency virus, acquired immunodeficiency syndrome, bone marrow, organ transplantation, malignancy, or previous varicella-zoster virus infection, could not be evaluated because of the limited number of cases. Third, the dose of prednisolone or methotrexate, the grade of CKD, and the history of herpes zoster vaccination could not be assessed because these data were not recorded in the JADER database or were missing. Herpes zoster vaccination is recommended for patients over 50 years of age [26]. Considering that the inactivated herpes zoster vaccine in Japan was approved in January 2020 and that the data in this study were from 2009 to 2021, the effect of the herpes zoster vaccination was unclear in this study [27].

## Conclusions

The findings of this study suggest that a tofacitinib dose of 5 mg/day may lower the risk of herpes zoster compared with 10 mg/day in patients with rheumatoid arthritis. Considering that the number of elderly people at high risk of herpes zoster has been increasing and that the onset of herpes zoster worsens prognosis in patients with rheumatoid arthritis, periodic evaluation of the possibility of reducing the dose of tofacitinib and recommendations for herpes zoster vaccination may improve the continuity of care for rheumatoid arthritis patients.
